# Botulinum toxin A for sialorrhea in adults with neurological disorders: a narrative review of aspiration pneumonia risk

**DOI:** 10.3389/fneur.2026.1851936

**Published:** 2026-07-17

**Authors:** Sultan Alzarouni, Krishab Sati, Prashanth Reddy, Mubasher A. Qamar

**Affiliations:** 1 Parkinson's Foundation Center of Excellence, King's College Hospital, King's College London, London, United Kingdom; 2 School of Medicine, The Royal College of Surgeons in Ireland (RCSI), Dublin, Ireland

**Keywords:** adults, aspiration pneumonia, botulinum toxin A, chronic hypersalivation, neurological disorders, sialorrhea

## Abstract

**Background:**

Aspiration pneumonia (AP), a serious condition resulting from the inhalation of foreign materials into the lungs, is a significant concern for patients with compromised swallowing mechanisms, especially those with neurological disorders. Sialorrhea (hypersalivation) exacerbates the risk of aspiration, and botulinum neurotoxin type A (BoNT-A) has been identified as a potential treatment to manage sialorrhea. However, the direct impact of BoNT-A on reducing AP risk remains inadequately explored, particularly in adult populations. BoNT-A works by inhibiting the release of acetylcholine at the neuromuscular junction, thereby reducing salivary gland stimulation and saliva production.

**Objective:**

This review aims to evaluate the current state of research on the use of BoNT-A for managing sialorrhea and its potential to reduce AP risk, with a specific focus on the adult population.

**Methods:**

A comprehensive review of existing literature was conducted, focusing on the efficacy of BoNT-A in treating sialorrhea in both pediatric and adult populations. Studies were assessed for their findings on the impact of BoNT-A on drooling and related complications, including the risk of AP. Meta-analyses and individual studies were reviewed to summarize the current knowledge and identify research gaps.

**Results:**

In pediatric patients, BoNT-A has been shown to effectively reduce sialorrhea, with several studies and meta-analyses demonstrating its efficacy in managing drooling associated with neurological conditions such as cerebral palsy. However, evidence linking BoNT-A directly to reduced AP risk is limited. In adults, while BoNT-A has been beneficial in managing sialorrhea associated with conditions such as Parkinson's disease and brain injuries, studies specifically exploring its impact on AP risk are lacking.

**Conclusion:**

Current evidence supports the efficacy of BoNT-A in managing neurological sialorrhea across both adult and pediatric populations. However, a critical gap exists in studies directly investigation whether BoNT-A reduces AP risk in adults with neurological disease.

## Introduction

Patients with neurological conditions such as stroke, Parkinson's disease (PD), amyotrophic lateral sclerosis (ALS), cerebral palsy (CP), motor neuron disease, and traumatic brain injury frequently experience complications related to bulbar dysfunction, dysphagia, and impaired secretion management. Among these complications, sialorrhea is particularly common (1) and represents a significant clinical burden (2–4). Sialorrhea may present as anterior droling, in which saliva escapes from the mouth and is often the more visible and socially distressing manifestation, or posterior drooling, in which pooled secretions spill over the tongue into the oropharynx, where it may be aspirated (3). Salivary pooling may arise from multiple contributing factors, including dysphagia, reduced level of consciousness, impaired orofacial motor control, and weakened swallowing function (1, 5, 6), and, in some cases, tracheostomy-related secretion retention (7). Normally, saliva in the hypopharynx triggers the swallowing reflex, but if this mechanism is impaired, saliva can accumulate and lead to coughing, gagging, respiratory congestion, or aspiration into the airway causing lower respiratory complications (8).

Saliva is primarily produced by the paired sublingual, parotid, and submandibular gland contributing the majority of resting and stimulated saliva. The submandibular glands produce approximately 65% of total salivary output, the parotid glands around 23%, and the sublingual glands about 4%, with the remaining 8% contributed by numerous minor salivary glands (9–11). Its secretion is tightly regulated by a predominantly autonomic, nerve-mediated reflex driven by gustatory, mechanical, olfactory, and thermal stimuli. Parasympathetic cholingergic signaling (mainly via M3 muscarinic receptors) is the principal driver of high-volume, watery saliva (85). Salivary composition and flow are dynamic, ranging from low resting rates to markedly increased stimulated secretion during feeding, and are influenced by circadian rhythms and central neural modulation (85). Beyond lubrication and digestion, it plays key roles in oral homeostasis, including buffering, antimicrobial defense, and mucosal protection, and serves as a biologically accessible fluid containing proteins, electrolytes, cellular components, and disease biomarkers (11).

Beyond its physical manifestations, sialorrhea substantially impairs quality of life and contributes to significant psychosocial distress (82). Oral health often deteriorates due to impaired secretion management and hygiene, leading to halitosis, perioral dermatitis, and oral discomfort (12, 13). Patients may further experience dehydration, difficulty eating and speaking, fatigue related to sleep disturbance, and reduced social participation (14, 15, 82). In severe cases, embarrassment, anxiety, depression, social isolation, and reduced self-esteem may occur (12, 16). The burden of sialorrhea also extends to caregivers, who frequently experience increased emotional and practical care demands (17, 82).

From a pathophysiological perspective, neurological sialorrhea is primarily related to impaired swallowing and reduced oral clearance rather than increased salivary production. Impaired bulbar function contributes to salivary pooling and reduced airway protection, which may be associated with increased aspiration risk in vulnerable neurological populations (18). In addition, reduced oral clearance and impaired oral hygiene may contribute to oropharyngeal bacterial colonization, which is considered a potential factor in aspiration-related infection risk (19, 20). The mechanisms underlying sialorrhea in neurological disorders are complex and multifactorial. Dysfunction or weakness of the oral and facial musculature, impaired coordination between orofacial and palatal-lingual muscles, reduced swallowing frequency, and weakened swallowing reflexes contribute to ineffective clearance of saliva from the oral cavity (10). Salivary accumulation may be associated with bacterial colonization and has been implicated in aspiration-related pulmonary complications, including aspiration pneumonia (AP), lung abscess formation, chronic pulmonary damage, and, in severe cases, respiratory failure (21, 22). AP is a serious respiratory infection that occurs when food, liquids, or saliva are inhaled into the lungs. This can trigger an inflammatory response due to the introduction of bacteria from saliva and secretions into the respiratory system.

Among these complications, AP represents a major clinical consequence of dysphagia and sialorrhea in neurological populations and is associated with significant morbidity and mortality. In the United States and United Kingdom, AP accounts for approximately 5%−15% of community-acquired pneumonia cases and is associated with a 30-day mortality rate approaching 21% (23). Neurological patients are particularly vulnerable, with consistently elevated AP risk reported across multiple conditions. In acute stroke populations, dysphagia-associated aspiration markedly increases the risk of pneumonia, with a pooled odds ratio of 9.60 in a recent systematic review and meta-analysis (101). The association is especially well documented in Parkinson's disease, where a recent meta-analysis reported an approximately threefold increased risk of AP compared with the general population (24, 81). Elevated AP rates have also been reported in ALS, progressive supranuclear palsy (PSP), stroke, multiple sclerosis (MS), and dementia populations (Table 1). In stroke populations specifically, reported AP incidence varies substantially across studies, ranging from approximately 7% in large cohort studies to over 20%−30% in more severe hospitalized cohorts, likely reflecting differences in stroke severity, patient selection, and diagnostic criteria [Table 1; (99, 102)]. Incidence estimates of approximately 13% in ALS and 24% in PSP have also been reported, while increased AP-related burden has been described in MS populations (25–27).

**Table 1:** Risk of aspiration pneumonia in neurological disorders.

Neurological condition	Reference	Study design/sample size	Type of measure	Key finding	Interpretation/limitation
Parkinson's disease	Chua et al. (24)	Meta-analysis of 13 studies; *n* = 541,785,587	Relative risk (RR)	RR = 3.30 for AP compared to controls	Strong population-level evidence supporting increased AP risk in PD; however, pooled administrative datasets introduce heterogeneity in AP definitions and study methodology
Parkinson's disease	Won et al. (50)	Retrospective cohort; *n* = 10,159	Incidence rate	3.01 vs 0.59 AP events/1,000 person-years	Supports substantially elevated AP incidence in PD populations
Parkinson's disease	Akbar et al. (51)	Retrospective cohort; *n* = 5,665,710	Incidence	3.6% vs 1% AP incidence	Large cohort study supporting increased AP burden in PD
Parkinson's disease	Di Luca et al. (52)	Retrospective cohort; *n* = 334,395	Adjusted odds ratio (AOR)	AOR 7.55 for adverse outcomes including AP	Suggests AP contributes significantly to poor clinical outcomes; AP not isolated as primary endpoint
Parkinson's disease	Fernandez et al. (89)	Retrospective cohort; *n* = 15,237	Relative risk (RR)	AP associated with highest mortality risk ratio (RR 1.58)	Highlights major prognostic impact of AP in PD
Parkinson's disease	Fujioka et al. (90)	Retrospective cohort; *n* = 136	Hospitalization frequency	AP represented 12.5% of hospitalizations	Small cohort size limits generalisability
Parkinson's disease	George et al. (91)	Retrospective cohort; *n* = 35,457	Odds ratio (OR)	OR 1.17 for AP development	More modest association compared to previous studies; possible methodological differences
Parkinson's disease	Guttman et al. (92)	Retrospective cohort; *n* = 15,304	Incidence	AP incidence rate 6.34	Supports elevated respiratory burden in PD
Parkinson's disease	Mahajan et al. (93)	Retrospective cohort; *n* = 3,015,645	Hospitalization prevalence	Pneumonia represented 6.3% of PD hospitalizations	Administrative data may overestimate hospitalization-related disease burden
Parkinson's disease	Wu et al. (94)	Retrospective case-control; *n* = 1,213	Hazard ratio (HR)	HR 2.45 for AP incidence	Suggests significantly increased longitudinal AP risk
Parkinson's disease	Chang et al. (53)	Retrospective cohort; *n* = 2,001	Hospitalization incidence	19% hospitalized with pneumonia	Reflects substantial respiratory morbidity in PD
Parkinson's disease	Akbar et al. (54)	Retrospective cohort; *n* = 1,087,667,580	Incidence	3.9% vs 0.69% AP incidence	Very large database study; findings strengthened by sample size but subject to coding heterogeneity
Parkinson's disease	Guneysel et al. (95)	Retrospective cohort; *n* = 76	Hospitalization cause	Infections accounted for 31.6% of admissions	Small sample size limits external validity
Multiple sclerosis	Harding et al. (27)	Retrospective cohort; *n* = 771,288	Adjusted odds ratio (aOR)	aOR 7.15 for AP-related mortality	Suggests AP significantly contributes to mortality burden in MS
Progressive supranuclear palsy	Tomita et al. (26)	Retrospective cohort; *n* = 90	Incidence	24% AP incidence	Supports high AP vulnerability in PSP populations
Progressive supranuclear palsy	Tomita et al. (26)	Retrospective cohort	Incidence	24% AP incidence	Limited participant data available
Amyotrophic lateral sclerosis	Sorenson et al. (25)	Retrospective cohort; *n* = 40	Incidence	13% AP incidence	Supports clinically significant AP burden in ALS
Amyotrophic lateral sclerosis	Pisa et al. (96)	Retrospective cohort; *n* = 262	Hospitalization cause	Pneumonia represented 14.2% of hospitalizations	Respiratory complications remain major contributor to hospitalization
Amyotrophic lateral sclerosis	Kurian et al. (55)	Retrospective cohort; *n* = 44	Mortality proportion	AP accounted for 11% of deaths	Small cohort size limits interpretation
Amyotrophic lateral sclerosis	Wolf et al. (97)	Prospective cohort; *n* = 200	Mortality proportion	Pneumonia represented 9.6% of deaths	Prospective design strengthens validity
Stroke	Katzan et al. (56)	Retrospective cohort; *n* = 14,293	Incidence	Pneumonia identified in 6.9% of stroke admissions	Demonstrates common post-stroke respiratory complication
Stroke	Finlayson et al. (102)	Retrospective cohort; *n* = 8,251	Incidence	Stroke-associated pneumonia in 7.1%	Consistent with previous stroke cohorts
Stroke	Wilson et al. (98)	Retrospective cohort; *n* = 183,976	Relative risk (RR)	Pneumonia-associated mortality RR = 2.0	Highlights prognostic significance of AP following stroke
Stroke	Nigus et al. (99)	Retrospective cross-sectional study; *n* = 242	Incidence	23,1% AP incidence	High AP incidence likely reflects hospitalized severe stroke population; single-center retrospective design limits generalisability
Stroke	Lidetu et al. (100)	Retrospective cohort; *n* = 568	Incidence	23,06% of AP incidence	Supports the high burden of post-stroke aspiration pneumonia
Dementia	Manabe et al. (57)	Meta-analysis; *n* = 79,956	Odds ratio (OR)	OR 2.15 for pneumonia-associated mortality	Suggests dementia substantially increases AP-related mortality risk
Dementia	Bosch et al. (58)	Prospective cohort; *n* = 120	Prognostic association	AP associated with increased mortality	Small prospective study supporting prognostic importance of AP

Given the multifactorial nature of sialorrhea, management typically requires a multidisciplinary approach combining non-pharmacological, pharmacological, and procedural strategies. Non-pharmacological conservative interventions form the cornerstone of initial management and include oral hygiene optimisation, behavioral and postural strategies during feeding and rest, dysphagia rehabilitation, secretion suctioning, and speech and language therapy support (28, 29). Additional input utilizing a multidisciplinary approach with speech and language therapists may support dysphagia management, while physiotherapy may assist with postural optimisation and respiratory management in patients (30, 31, 73).

Pharmacological management primarily relies on anticholinergic agents, including glycopyrrolate, scopolamine, and atropine, which reduce salivary secretion through muscarinic receptor inhibition within the salivary glands (10, 32, 33). Although effective, their use is often limited by systemic adverse effects such as urinary retention, constipation, blurred vision, cognitive impairment, and excessive dryness (34). Consequently, safer and more targeted interventions are frequently preferred.

Botulinum toxin (BoNT) injections have emerged as a major therapeutic option for neurological sialorrhea (79). BoNT-A acts by inhibiting acetylcholine release at the presynaptic neuromuscular junction within salivary glands, thereby reducing salivary production without inducing systemic anticholinergic effects (33). Injection protocols targeting all four major salivary glands have demonstrated substantial reductions in salivary production (75).

BoNT-A offers a targeted approach for reducing salivary secretion by inhibiting acetylcholine release at the neuromuscular junction within salivary glands, avoiding systemic anticholinergic effects (74). This is particularly relevant in older neurological patients with frailty, cognitive impairment, comorbidities, and polypharmacy (34, 35, 84). Additionally, while surgical interventions such as salivary duct ligation or gland excision may provide symptomatic benefit, they are invasive, require general anesthesia, and are associated with higher complication rates, limiting their suitability for routine use in medically complex neurological populations (10, 73, 74).

The aim of this narrative review is therefore to critically evaluate the evidence on BoNT-A in the management of sialorrhea and its potential impact on AP risk in adult neurological populations, while highlighting key gaps in the current literature.

## Methodology

A comprehensive literature review was conducted to explore the utilization of botulinum toxin A (BoNT-A) in managing AP risk in adults with neurological disorders. This review was conducted as a structured narrative review. No formal meta-analysis was performed because of heterogeneity in study design, outcomes, and reporting. Elements of a structured search and transparent reporting were adapted from PRISMA principles to enhance clarity, but key features of a systematic review (such as protocol registration and exhaustive, predefined outcome synthesis) were not applied.

**Search strategy:** A structured search was performed using electronic databases, including PubMed, Scopus, Google Scholar, and Embase, to identify peer-reviewed studies published in English. The search terms included combinations and synonyms of the following keywords: “Botulinum Toxin A,” “aspiration pneumonia,” “neurological disorders,” “sialorrhea,” “dysphagia,” “oropharyngeal dysfunction,” and “intervention therapy.” Boolean operators (AND, OR) were used to refine search results and ensure inclusivity. This review was conducted as a structured narrative review.

Efforts were made to maintain a structured and transparent approach to study identification and synthesis, adapted from PRISMA principles for a narrative review to provide a broad, transparent, and integrative overview.

Studies were selected based on predefined inclusion and exclusion criteria:

- **Inclusion criteria:**

° Studies evaluating the efficacy and safety of BoNT-A in managing aspiration pneumonia risk.
° Studies involving adult patients with neurological disorders (e.g., PD, ALS, stroke, MS).
° Studies involving pediatric patients with neurological sialorrhea (e.g., CP and neurological dysfunction) when they provided relevant background, foundational evidence, or additional context regarding efficacy and safety.
° Clinical trials, systematic reviews, meta-analyses, and cohort studies published from the year 2000 onwards reporting clinically relevant outcomes related to drooling control, swallowing function, respiratory complications, or AP.
° Published in the English language.
° Full text and data availability.
° Open-access sources only.

- **Exclusion criteria:**

° Studies focusing solely on pediatric populations.
° Articles not available in English.
° Case reports and conference abstracts lacking sufficient data.
° Studies evaluating other botulinum toxin formulations (e.g., BoNT-B, BoNT-C, BoNT-E, BoNT-F, or experimental neurotoxins) that do not involve BoNT-A specifically.

**Study selection and data extraction:** titles and abstracts of retrieved articles were screened for relevance, followed by full-text evaluation. Two independent reviewers assessed the studies to ensure reliability and minimize bias. Any discrepancies were resolved through discussion with a third reviewer. Extracted data included study design, population characteristics, intervention details (dosage, administration site), outcomes measured (improvement in swallowing function, reduction in aspiration risk), and reported adverse effects.

**Quality assessment:** the quality of selected studies was appraised using appropriate tools: the Cochrane Risk of Bias Tool for randomized controlled trials, the Newcastle-Ottawa Scale for observational studies, and AMSTAR-2 for systematic reviews. Studies meeting high methodological standards were prioritized in the discussion and synthesis of findings.

**Data synthesis:** findings were synthesized narratively due to the heterogeneity of study designs and outcome measures. Key themes, including efficacy, safety, and limitations of BoNT-A as a therapeutic approach, were identified and discussed in relation to current clinical practice.

This methodological approach ensures a comprehensive and unbiased review of the literature, providing valuable insights into the role of BoNT-A in reducing aspiration pneumonia risk among adult patients with neurological disorders.

### Review of existing evidence

Botulinum toxin type A (BoNT-A) is widely used to manage sialorrhea in patients with neurological disorders. Its efficacy and safety in pediatric populations, especially in children with cerebral palsy (CP) and other neuromuscular conditions, have been demonstrated across multiple studies and clinical trials, showing reductions in sialorrhea and related complications (36–39, 77, 78). These findings establish BoNT-A as a therapeutic option for children experiencing neurological sialorrhea.

A substantial body of evidence supports the use of BoNT-A for sialorrhea in adults with neurological disorders including Parkinson's Disease (PD), stroke, amyotrophic lateral sclerosis (ALS), and traumatic brain injury (80, 83). Although similar pathophysiological mechanisms underlie sialorrhea in both adults and children, the adult evidence base regarding BoNT-A's specific role in preventing AP remains limited and has not been comprehensively explored. IncobotulinumtoxinA is currently the only BoNT formulation specifically approved for chronic neurological sialorrhea in adults in several regions (3).

Clinical studies assessing BoNT-A in adult neurological patients have reported reductions in saliva production and improvements in functional measures (33, 83). For example, a meta-analysis in adult neurological cases documented significant improvements in drooling severity and frequency, as measured by the Drooling Severity and Frequency Scale (DSFS) (40). Various studies demonstrate the efficacy of BoNT-A for patients with PD, stroke, traumatic brain injury (TBI), and tetraplegia, with reported functional benefits and reductions in salivation (41–46). Additionally, in people with severe acquired brain injury, salivary gland BoNT-A is an effective treatment for sialorrhea that significantly lowers DSFS scores and reduces chest infection frequency, with the greatest benefit seen in those receiving repeated injections and higher doses (47). The SIAXI trial (NCT02091739), a randomized, double-blind, placebo-controlled study, evaluated the safety and efficacy of 100 units of incobotulinumtoxinA in adults with neurological sialorrhea showing a significant reduction in unstimulated salivary flow at week 4 with 100 units of incobotulinumtoxinA, supporting adult efficacy for chronic sialorrhea, but it did not primarily test for AP reduction (75, 86).

BoNT-A appears effective and safe for symptomatic management of sialorrhea. However, study heterogeneity is substantial, including differences in neurological populations, disease severity, injection techniques, dosing, and outcome measures (Table 2).

**Table 2:** Botulin toxin for sialorrhea issues in adult and pediatric neurological patients.

Neurological population	Reference	Study design	Participants (*n*)	Botulinum toxin regimen	Primary outcome/ measure	Key findings	Interpretation	Limitations
Adult PD	Chinnapongse et al. (59)	Clinical trial	54	1,500; 2,500 or 3,500 U BoNT-B	Drooling frequency	Significant improvement at 4 weeks post-injection	Supports short-term efficacy of BoNT-B for PD-related sialorrhea	Short follow-up duration
Adult PD	Lagalla et al. (60)	Clinical study	36	4,000 U BoNT-B	Drooling score/global improvement	Significant reduction in sialorrhea one month post-treatment	Supports efficacy of BoNT-B in PD	No long-term outcomes
Adult PD	Mancini et al. (44)	Clinical study	20	450 U BoNT-A	Drooling reduction	Reduction in drooling severity observed	Supports efficacy in PD	Small cohort
Adult PD	Narayanaswami et al. (33)	Clinical study	10	100 U BoNT-A	Drooling efficacy	No statistically significant improvement observed	Highlights variability in treatment response	Very small sample size
Adult PD	Ondo et al. (61)	Clinical study	16	1,000 U BoNT-B	Drooling rating scales	Improvement in global impression and drooling scales	Supports efficacy of BoNT-B	Limited sample size
Adult PD	Dogu et al. (42)	Clinical study	15	15 U BoNT-A per parotid gland	Drooling improvement	Improvement observed within first week	Suggests rapid therapeutic effect	Small cohort
Adult PD	Nobrega et al. (62)	Clinical study	16	125 U BoNT-A (Dysport^®^)	Sialorrhea severity	Decrease in sialorrhea observed	Supports efficacy in PD	Limited sample size
Adult PD	Santos Junior et al. (46)	Meta-analysis (13 RCTs)	13 RCTs	Different doses of BoNT-A and B	Salivary flow and drooling severity	Reduction in drooling severity/frequency; no difference between toxin types	Supports efficacy across BoNT formulations	Heterogeneity across included trials
Adult PD	Yang et al. (40)	Meta-analysis	259	N/A	DSFS reduction	Significant reduction in drooling severity/frequency	Reinforces overall efficacy findings	Limited adult-specific AP data
Adult ALS	Jackson et al. (87)	Placebo-controlled study	20	2,500 U BoNT-B	Global impression of improvement	Improvement in 82% vs 38% placebo at 2 weeks	Demonstrates symptomatic benefit in ALS	Limited participant number
ALS	Weikamp et al. (63)	Clinical study	10	20 or 50 MU BoNT-A (Dysport^®^ 200 U/mL)	Swallowing function	Swallowing function preserved after treatment	Supports safety profile regarding dysphagia	Did not assess AP outcomes
Adult PD and ALS	Guidubaldi et al. (64)	Comparative study	27	250 U BoNT-A vs 2,500 U BoNT-B	Drooling improvement and onset latency	Both treatments improved symptoms; shorter latency with BoNT-B	Suggests both serotypes may be effective	Small sample size
Adult PD and ALS	Moller (80)	Clinical study	15	15–40 U BoNT-A (Botox^®^)	Drooling and salivary flow	Maximal drooling reduction of 40% at 2 weeks	Demonstrates moderate symptomatic improvement	Small heterogeneous cohort
Mixed adult neurological populations	Jost et al. (86)	Randomized placebo-controlled trial (SIAXI)	184	75 or 100 U incobotulinumtoxinA (Xeomin^®^)	Salivary flow rate	Significant reduction, particularly with 100 U	Strong evidence supporting BoNT-A efficacy in adults	Focused on surrogate outcomes rather than AP
Adult	Lipp et al. (88)	Dose-escalation study	32	18.75; 37.5 and 75 MU BoNT-A	Global improvement	Overall symptomatic improvement observed	Suggests dose responsiveness	Heterogeneous population
Adult neurological disorders	Mazlan et al. (45)	Comparative dosing study	30	50, 100 or 200 U BoNT-A (Dysport^®^)	Salivary reduction / drooling severity	All doses reduced saliva; greatest effect with 200 U	Suggests higher doses may improve efficacy	No significant inter-dose statistical difference
Adult neurological disorders	Martinez-Poles et al. (65)	Clinical study	36	43.2 U inco-A per parotid gland	DSFS improvement	Significant improvement in drooling scales	Supports symptomatic benefit	No respiratory endpoints evaluated
Adult tetraplegic patients with brain injury	Ko et al. (41)	Clinical study	8	100 U BoNT-A (Botox^®^)	Drooling severity/frequency	Improvement at 3 weeks and 3 months	Suggests sustained benefit	Very small sample size
Adult and children	Raval and Elliott (66)	Retrospective review	12	BoNT-A	Hospitalizations / pulmonary infections	Reduction in respiratory hospitalizations and infections	Suggests possible respiratory benefit	Retrospective design and small sample
Adult and children with neurological disorders	Yu et al. (38)	Meta-analysis	981	Different doses of BoNT-A and B	Drooling severity/frequency	Reduced drooling severity, saliva weight, and improved global impression	Strong evidence for symptomatic efficacy	Considerable heterogeneity among studies
Neuroleptic-induced sialorrhea	Steinlechner et al. (67)	Clinical study	9	2,500 U BoNT-B (Neurobloc^®^)	Duration of improvement	Symptom improvement lasting 8–16 weeks	Suggests sustained efficacy	Very small sample size
Children with CP	Silva et al. (74)	Meta-analysis	141	BoNT-A	Drooling improvement	Significant symptomatic improvement	Supports efficacy in CP	Focused primarily on pediatric populations
Children with CP	Hung et al. (79)	Meta-analysis	827	Dysport^®^ and Botox^®^	Drooling improvement	Significant efficacy lasting 8–12 weeks	Strong pediatric evidence base	Heterogeneous dosing protocols
Children with CP	Reid et al. (68)	Clinical study	24	25 U BoNT-A	Drooling severity	Maximum improvement at 1 month, sustained at 6 months	Suggests sustained medium-term efficacy	Small cohort
Children with CP	Jongerius et al. (8)	Clinical study	45	15–20 U/submandibular gland BoNT-A (Botox^®^)	Drooling reduction	Maximum effect between 2–8 weeks	Supports efficacy in CP-related drooling	Limited long-term follow-up
Children – mixed neurological disorders	Ha et al. (69)	Clinical study	606	N/A	Sialorrhea improvement	Improvement in drooling symptoms observed	Supports pediatric efficacy	No control group and limited methodological detail
Children with neurological disorders	Jeung et al. (70)	Clinical study	17	0.5 U/kg BoNT-A	Drooling frequency/severity	Improvement sustained up to 9 months	Suggests prolonged benefit	Small sample size
Children	Pena et al. (71)	Retrospective review	36	BoNT-A	Respiratory hospitalization	88% drooling improvement; respiratory hospitalizations reduced by 56.4%	Suggests potential respiratory benefit	Retrospective design
Children with neurological dysfunction	Gubbay and Marie Blackmore (39)	Clinical study	17	BoNT-A	Severity/frequency/impact	Reduction in drooling burden	Supports quality-of-life improvement	Small participant number
Children	Oad et al. (72)	Meta-analysis	131	Different doses of BoNT-A	Drooling severity	Overall improvement in drooling severity	Reinforces pediatric efficacy findings	Heterogeneity in included studies

PD, Parkinson's Disease; ALS, amyotrophic lateral sclerosis; CP, cerebral palsy; RCT, randomized controlled trial

While most studies report positive outcomes, not all demonstrate uniform efficacy. For example, a randomized cross-over trial showed that BoNT-A did not significantly reduce saliva weight or drooling severity in a small PD cohort compared to placebo, highlighting variability in treatment response, suggesting possible effects of dose, formulation, or study heterogeneity (33). Conversely, other studies and meta-analyses have reported clinically meaningful reductions in drooling outcomes (38, 40, 44, 60, 65).

Importantly, most studies focus on surrogate outcomes such as drooling scales or salivary flow rather than clinically meaningful endpoints such as aspiration pneumonia, hospitalization, or mortality. Evidence regarding respiratory outcomes remains limited and largely indirect; however, a retrospective study reported a significant reduction in chest infection frequency after salivary gland BoNT-A in adults with severe acquired brain injury (47). Moreover, the potential for BoNT-A to help reduce the risk of aspiration pneumonia (AP) in the adult population has not been comprehensively investigated (45, 48).

Overall, evidence supporting the efficacy of BoNT-A for reducing sialorrhea is strong, AP specific outcomes are lacking in both pediatric and adult populations. Adult data on sialorrhea treatment are not sparse overall; rather the key gap is the absence of clinically meaningful respiratory endpoints in adults with neurological disorders despite higher clinical complexity and greater burden of AP risk (3, 38). Table 2 distinguishes key clinical studies that have investigated BoNT-A treatment across both adult and pediatric populations. This review therefore highlights the need for further high-quality studies evaluating clinically relevant respiratory outcomes, including AP, in adult neurological populations treated with BoNT-A.

## Discussion

This narrative review confirms that BoNT-A is an effective symptomatic treatment for neurological sialorrhea and that its use is supported by a well-established body of clinical evidence. Across studies, BoNT-A consistently reduces drooling severity and salivary flow, and current practice increasingly favors ultrasound-guided injection into the bilateral parotid and submandibular glands (48). Four-gland injection protocols appear to provide the most reliable symptomatic benefit, with selected studies reporting high response rates, including efficacy rates of 89% and 95% (49, 76). These findings reinforce the role of BoNT-A as a practical, localized, and generally well-tolerated intervention for patients whose sialorrhea is not adequately controlled with conservative or pharmacological measures. The critical question this review addresses, is whether these established symptomatic benefits translate into meaningful reductions in AP risk, and the answer, based on current evidence, remains uncertain.

However, the strongest and most consistent evidence relates to improvement in drooling rather than prevention of aspiration-related complications. Most studies have used surrogate outcomes such as drooling frequency, secretion volume, or global clinical impression, and comparatively few have evaluated AP, hospitalization, ICU admission, or mortality. This evidence gap is clinically important; as outlined in the introduction, adults with neurological disease carry a substantially elevated burden of AP-related morbidity and mortality, and BoNT-A has been proposed as a strategy to address this through secretion reduction and improved airway protection. At present, the evidence base does not allow firm conclusion about whether BoNT-A meaningfully alters these downstream outcomes.

The relationship between sialorrhea and AP is complex and cannot be explained solely by salivary volume. Multiple interacting factors, including swallowing coordination, level of consciousness, oropharyngeal bacterial colonization, and aspiration reflex integrity, determine AP risk independently of secretion volume.

Many available studies are small, heterogeneous, and focused on short-term symptomatic outcomes rather than longer-term clinical events. Differences in dose, gland selection, injection technique, imaging guidance, outcome definition, and follow-up period make cross-study comparisons difficult. AP itself is not always uniformly defined, and outcome assessment may be influenced by local diagnostic practice, which further weakens comparability. These issues limit the certainty with which BoNT-A can be judged to modify clinically important respiratory events.

The relevance of BoNT-A may also vary according to the underlying neurological condition and the severity of bulbar impairment. In disorder with potentially evolving or partially reversible swallowing dysfunction, secretion reduction may have different implications than in progressive neurodegenerative disease, where dysphagia and airway protection may continue to worsen despite treatment. This heterogeneity argues against overgeneralising findings across diagnostic groups and supports the need for condition-specific studies rather than broad assumptions about universal benefit (24, 50). It is therefore more accurate to view BoNT-A as a treatment with established symptomatic value and uncertain respiratory-modifying effects.

From a clinical perspective, BoNT-A remains a useful part of multidisciplinary sialorrhea management, particularly for patients who do not tolerate anticholinergic therapy or in whom systemic side effects are problematic. The treatment is attractive because it is targeted, repeatable, and generally associated with an acceptable safety profile. Nevertheless, clinicians should integrate BoNT-A into a broader care plan that includes swallowing assessment, oral hygiene, nutritional support, and respiratory risk management, especially in patients with significant dysphagia or recurrent chest infections. This is particularly important because symptomatic improvement alone does not guarantee reduction in aspiration-related morbidity.

Future research should move beyond surrogate endpoints and prioritize clinically meaningful outcomes. Prospective studies should examine AP, hospitalization, antibiotic use, ICU admission, mechanical ventilation, nutritional status, functional outcomes, quality of life, and mortality, ideally using standardized outcome definitions and longer follow-up periods. Further stratification by swallowing function, drooling pattern, and neurological diagnosis would help clarify which patients are most likely to benefit from treatment. Until such data is available, the benefit of BoNT-A should be described primarily in terms of drooling reduction, with any potential impact on AP regarded as plausible but unproven.

Overall, this review supports BoNT-A as an effective intervention for neurological sialorrhea, but the evidence remains insufficient to conclude that it reduces AP or other major respiratory outcomes. The main value of the current literature lies in confirming symptomatic efficacy, while also highlighting a clear gap between improvement in drooling and proof of downstream clinical benefit. Addressing this should be a priority for future research evaluating clinically meaningful respiratory outcomes in neurological sialorrhea.

## Limitations

This review has several limitations that should be acknowledged. First, as a narrative review, it is inherently subject to selection and interpretation bias and does not follow a predefined systematic methodology for study identification, screening, or inclusion. Although efforts were made to include representative and clinically relevant studies, incomplete literature capture and subjective selection of evidence cannot be excluded.

Second, the available literature is highly heterogeneous in terms of patient populations, neurological conditions, study designs, outcome measures, and follow-up durations, which limits comparability across studies. In addition, variability in the definition and reporting of aspiration pneumonia further complicates synthesis and interpretation.

Third, most available evidence focuses on surrogate outcomes such as drooling severity rather than clinically meaningful endpoints, including aspiration pneumonia incidence, respiratory hospitalization, or mortality. As a result, the true impact of botulinum toxin type A on pulmonary outcomes remains uncertain.

Finally, adult-specific data remain limited and are largely derived from observational studies, which reduces the overall strength of evidence. Publication bias may also have contributed to an overrepresentation of positive findings in the literature.

## Conclusion and future research directions

BoNT-A is an established and effective treatment for neurological sialorrhea, with symptomatic benefits well-supported across adult and pediatric populations. As described in this review, sialorrhea is a key modifiable risk factor for AP in adults with neurological disease, and there is a compelling rationale to evaluate whether BoNT-A's symptomatic benefits extend to reductions in AP incidence and related respiratory morbidity. Future research should prioritize longitudinal observational and interventional studies to evaluate the effectiveness of BoNT-A in reducing aspiration pneumonia risk in adult populations with neurological disease.
